# Rice Protein as an Alternative Ingredient for Sustainable Food Formulations: Nutritional Quality, Functional Properties, Health-promoting Potential, and Emerging Applications

**DOI:** 10.1007/s11130-026-01561-6

**Published:** 2026-08-24

**Authors:** Silvia Leticia Rivero Meza, Larissa Alves Rodrigues, Sonia Medina, Isabel Egea, Patricia Sinnecker, Mauricio de Oliveira

**Affiliations:** 1 Present Address: Life and Environmental Area, Caxias do Sul, State University of Rio Grande do Sul, Caxias do Sul, Rio Grande do Sul 95010-005 Brazil; 2 Present Address: Department of Agroindustry Science and Technology, Federal University of Pelotas, Pelotas, Rio Grande do Sul 96010-900 Brazil; 3 Present Address: Laboratorio de Fitoquímica y Alimentos Saludables (LabFAS), CSIC, CEBAS, Campus Universitario de Espinardo, Edificio 25, Murcia, 30100 Spain; 4 Present Address: Department of Stress Biology and Plant Pathology, CEBAS-CSIC, Espinardo-Murcia, 30100 Spain; 5 Present Address: Department of Pharmaceutical Sciences, Pharmaceutical Inputs and Food Sector, Federal University of São Paulo, Diadema, São Paulo 09913-030 Brazil

**Keywords:** Rice protein, Hypoallergenic proteins, Functional foods, Bioactive peptides, Preventive nutrition, Sustainable food systems

## Abstract

The growing demand for sustainable and nutritionally adequate protein sources has intensified interest in alternative plant proteins capable of supporting future food systems. However, many conventional plant proteins still present limitations related to allergenicity, digestibility, sensory quality, and functional performance, highlighting the need for novel sustainable ingredients. In this context, rice protein has emerged as a promising alternative due to its hypoallergenic nature, gluten-free composition, relatively high digestibility, suitability for specialized dietary applications, favorable techno-functional characteristics, and compatibility with sustainable food production systems. This review provides an integrated perspective on rice protein by critically discussing its nutritional quality, techno-functional properties, health-promoting potential, emerging food applications, and contribution to sustainable food systems, thereby addressing knowledge that has remained fragmented across previous reviews. Recent scientific studies addressing protein composition, digestibility, bioactive peptides, processing technologies, and applications in plant-based foods and nutraceutical systems were critically analyzed. Current evidence demonstrated that rice proteins and rice-derived peptides exhibit antioxidant, antihypertensive, anti-inflammatory, antidiabetic, and immunomodulatory activities, while recent advances in protein modification technologies have significantly improved solubility, emulsification, gelation, and structuring properties. Furthermore, the valorization of rice bran and broken rice supports circular economy strategies and sustainable food production. In this way, rice protein represents a strategic ingredient for the development of next-generation functional foods and sustainable health-oriented dietary systems.

**Supplementary Information:**

The online version contains supplementary material available at https://doi.org/10.1007/s11130-026-01561-6.

## Introduction

The growing global population, projected to reach nearly 9.7 billion by 2050, together with rapid urbanization and increasing pressure on natural resources, has intensified the search for sustainable and nutritionally adequate protein sources [1–3]. Traditional animal-based protein production systems, although nutritionally valuable, are associated with high greenhouse gas emissions, excessive freshwater consumption, biodiversity loss, and extensive land use, contributing significantly to environmental degradation and climate change [3, 4]. In this context, the global “protein transition” has emerged as a critical strategy to support sustainable diets, food security, and climate-smart food systems by reducing dependence on animal-derived proteins and promoting alternative protein sources [5, 6].

**Fig. 1:**
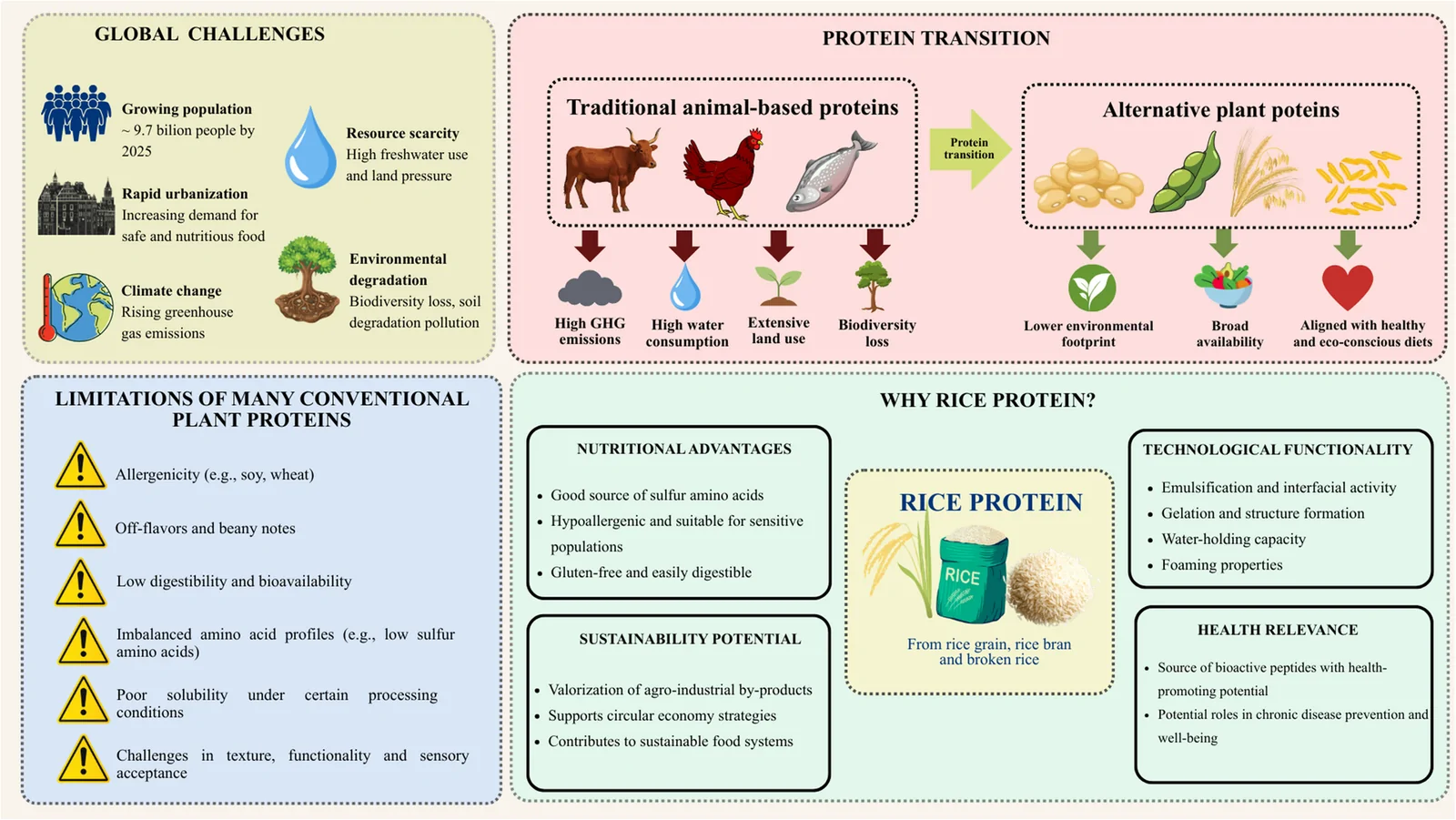
Conceptual framework illustrating the role of rice protein in sustainable and health-oriented food systems

Plant-based proteins have gained increasing attention due to their lower environmental footprint, broad availability, and alignment with consumer demand for healthier and more sustainable foods [2, 7, 8]. Proteins from soy, pea, wheat, oat, fava bean, and cereals have been widely incorporated into plant-based meat analogues, dairy alternatives, protein-enriched beverages, and functional foods [6, 8]. However, many plant proteins still exhibit important limitations, including allergenicity, off-flavors, low digestibility, imbalanced amino acid composition, poor solubility, and challenges related to texture and sensory acceptance [2, 5–7]. These limitations highlight the need for alternative plant proteins with improved nutritional and functional properties. The main drivers of the sustainable protein transition, the limitations of conventional plant proteins, and the emerging role of rice protein in next-generation food systems are summarized in Fig. 1.

Among emerging cereal proteins, rice protein has attracted growing scientific and industrial interest due to its unique nutritional and techno-functional properties. Rice is one of the world’s major staple crops and generates large amounts of milling by-products, including rice husk (approximately 20% of grain weight), rice bran (approximately 11%), and broken rice (approximately 10–15%), which represent valuable and underutilized sources of proteins and bioactive compounds [9, 10]. Rice protein is recognized for its hypoallergenic nature, gluten-free composition, relatively high digestibility, neutral flavor profile, and suitability for specialized dietary applications, including infant, elderly, and clinical nutrition [2]. In addition, rice-derived proteins support circular economy strategies through the valorization of agro-industrial side streams and the development of sustainable functional ingredients [11].

Rice protein also exhibits favorable techno-functional properties, including emulsification, water-holding capacity, gelation, and foaming, supporting its application in plant-based beverages, meat analogues, bakery products, and nutraceutical systems. Recent advances in food processing and protein modification technologies have further improved the functionality, digestibility, and bioactivity of rice proteins and rice-derived peptides [5, 11–14]. Nevertheless, its broader application remains limited by low lysine content, reduced solubility under certain processing conditions, lower commercial exploitation than soy and pea proteins, and the scarcity of clinical and industrial-scale studies evaluating long-term health and technological performance [2, 7].

Despite the growing body of literature on rice proteins, most published reviews have focused on specific aspects, including protein composition, extraction technologies, physicochemical characteristics, functional properties, emulsification behavior, protein modification strategies, industrial applications, and bioactive peptides. Consequently, current knowledge remains fragmented across nutritional, technological, health, and sustainability perspectives. Limited attention has been devoted to integrating these complementary dimensions within the broader context of sustainable food systems, where nutritional quality, preventive nutrition, health-promoting effects, techno-functional performance, circular bioeconomy opportunities, and emerging food applications should be considered simultaneously. Such integration has become increasingly important as plant protein research evolves from ingredient characterization toward the development of sustainable, resilient, and health-oriented food systems.

Therefore, this review aims to provide an integrated perspective on rice protein by critically discussing its nutritional quality, health-promoting potential, technological functionality, sustainability dimensions, and emerging food applications while identifying current limitations, knowledge gaps, and future research opportunities. Unlike previous reviews that primarily addressed individual aspects of rice protein, the present review integrates these complementary dimensions within a single framework, providing a broader perspective on the role of rice protein in sustainable and health-oriented food systems and highlighting future research priorities.

## Procedure and Data Analysis

The Procedure and Data Analysis section is described in Supplementary Material 1.

### Rice Protein in the Context of Sustainable Food Systems

The transition toward more sustainable food systems requires not only the diversification of protein sources but also the development of strategies capable of improving resource efficiency and reducing food losses throughout agricultural production chains. In this context, rice protein is particularly relevant because it can be recovered from abundant rice-processing side streams that are often underutilized despite their considerable nutritional and economic value. The recovery and utilization of proteins from rice-derived fractions contribute to the transformation of low-value biomass into value-added food ingredients, supporting more circular and resource-efficient production systems [15, 16].

The rice milling industry generates large quantities of by-products, including rice bran, broken rice, and defatted rice bran, which are rich in proteins, dietary fiber, minerals, and bioactive compounds that can be valorized through food processing technologies [15]. Historically, these fractions have been directed to low-value applications, such as animal feed, or remain underutilized. Their conversion into functional food ingredients offers an opportunity to increase the value of rice production while reducing material losses throughout the supply chain [13, 15].

From a circular bioeconomy perspective, rice protein valorization promotes the efficient use of agricultural resources and reduces waste generation. The incorporation of rice-derived proteins into food systems extends the value of rice raw materials, supports sustainable ingredient supply chains, and contributes to more resource-efficient food production [15, 16].

Recent advances in processing technologies have enhanced the sustainable utilization of rice-derived protein ingredients. Micronization, enzymatic modification, and protein fractionation improve protein recovery, stability, and industrial applicability, facilitating their incorporation into value-added food products while supporting sustainability goals [16–18]. These advances contribute to both environmental and economic sustainability in modern food systems.

**Fig. 2:**
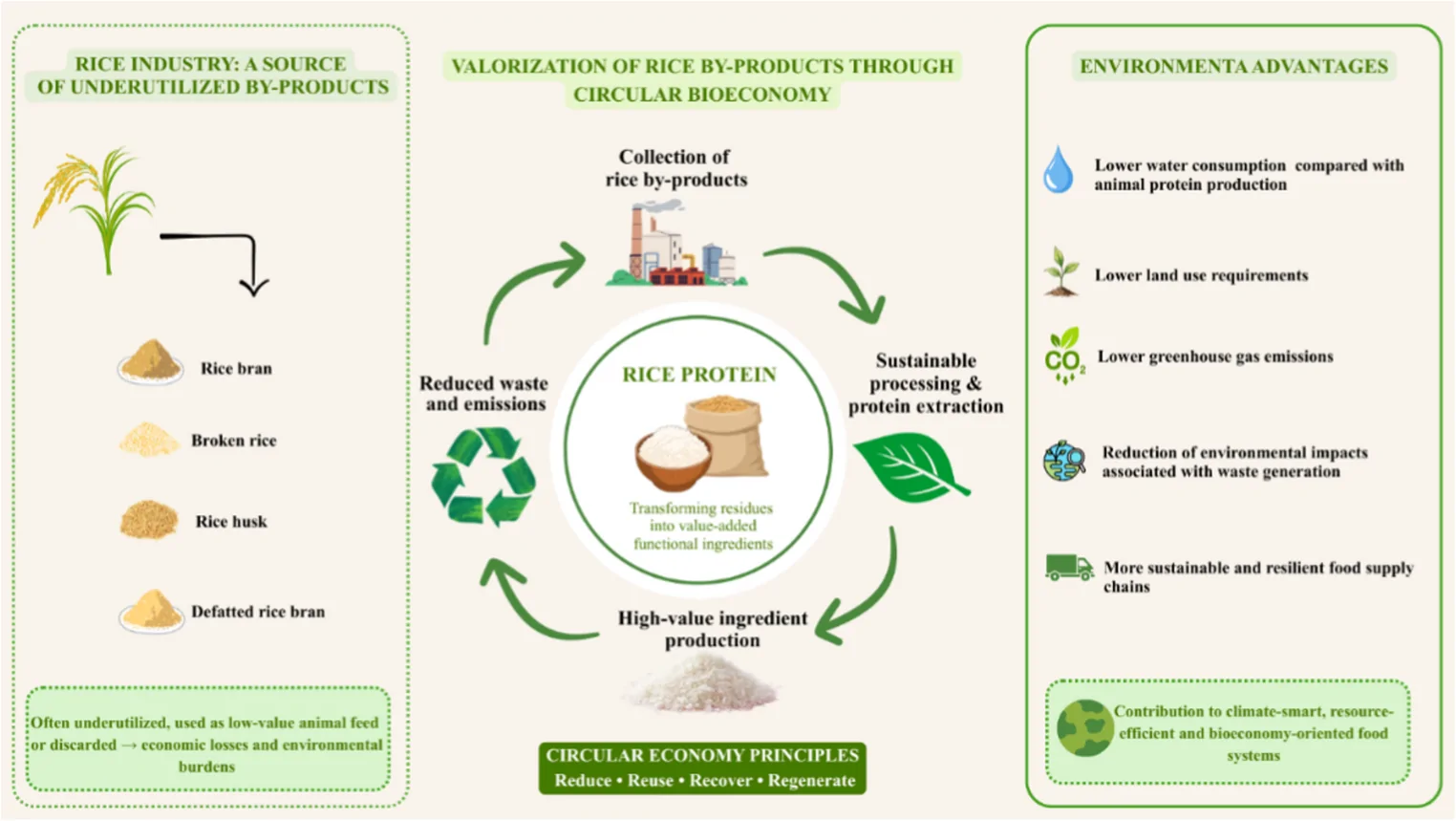
Sustainability framework highlighting the valorization of rice by-products and the contribution of rice protein to circular and resilient food systems

The sustainability relevance of rice protein extends beyond ingredient recovery and waste reduction. Its incorporation into food systems supports resilient supply chains by promoting the use of locally available resources and agro-industrial side streams, thereby reducing environmental impacts and contributing to long-term food security [13, 15]. Thus, rice protein represents not only a functional food ingredient but also a component of a broader sustainability framework based on circular bioeconomy, resource efficiency, by-product valorization, and food system resilience. These sustainability aspects are summarized in Fig. 2.

### Nutritional Relevance of Rice Protein for Human Nutrition

Beyond serving as a protein source, rice protein has gained attention due to its high digestibility, low allergenic potential, gluten-free nature, and suitability for specialized nutrition, including infant, elderly, sports, and clinical applications. Growing interest in preventive nutrition has further stimulated research on the potential of rice proteins and their derived compounds to promote health and reduce chronic disease risk factors. Therefore, evaluating the nutritional relevance of rice protein requires considering not only its protein quality and dietary suitability but also its contribution to health-promoting dietary strategies [13, 14]. The following sections discuss its protein quality, hypoallergenic and gluten-free characteristics, and relevance to preventive nutrition.

### Protein Quality

The nutritional quality of rice protein is closely related to its amino acid composition and digestibility. Rice proteins are mainly composed of glutelin, followed by prolamin, albumin, and globulin, with glutelin accounting for approximately 60–80% of total protein [19]. Rice protein contains relatively high levels of sulfur-containing amino acids, particularly cysteine and methionine, which are often limited in legume proteins [14]. Although lysine is the primary limiting amino acid, rice protein generally exhibits a more balanced amino acid profile and higher digestibility than many cereal proteins. Its complementary amino acid profile enhances nutritional quality when combined with plant proteins such as pea or soy, making it particularly suitable for balanced plant-based formulations [20]. Furthermore, favorable digestibility and protein quality support its application in diversified plant-based diets.

From a nutritional perspective, digestibility and amino acid bioavailability are key determinants of protein quality and physiological utilization. Rice protein is recognized for its relatively high digestibility and favorable gastrointestinal tolerance compared with wheat proteins and some legume proteins containing antinutritional factors [18]. However, digestibility varies according to protein source, extraction method, processing conditions, and residual antinutritional compounds, emphasizing the influence of processing on nutritional quality. Rice protein is also naturally gluten-free and has low allergenic potential, making it suitable for specialized diets, including those for individuals with celiac disease, food allergies, or digestive sensitivities [17]. These characteristics, together with its mild sensory profile and neutral flavor, have expanded its application in infant formulas, elderly nutrition, sports supplements, and clinical nutrition products while improving consumer acceptance of plant-based foods. The combination of nutritional quality, low allergenicity, and sensory neutrality provides an important advantage for its incorporation into food products.

Recent studies have shown that processing technologies significantly influence the digestibility and nutritional functionality of rice proteins. Enzymatic hydrolysis, fermentation, ultrasonication, and controlled protein modification improve solubility, peptide release, amino acid accessibility, and gastrointestinal digestibility while reducing protein aggregation [17, 19]. These approaches may also increase protein bioavailability and promote the generation of bioactive peptides with antioxidant, antihypertensive, and anti-inflammatory activities [17]. However, their effectiveness depends on processing conditions, protein source, and operational parameters, limiting direct comparisons among studies. Overall, these findings indicate that rice protein is not only a source of dietary amino acids but also a promising platform for multifunctional ingredients with nutritional, physiological, and technological benefits. Nevertheless, further clinical and long-term human studies are needed to confirm the health-promoting effects of rice-derived peptides under real dietary conditions.

### Hypoallergenic and Gluten-free Potential

The hypoallergenic nature of rice protein is a major nutritional advantage. Unlike soy and dairy proteins, two of the most common food allergens, rice protein exhibits low immunogenicity and excellent tolerability, making it suitable for sensitive populations and medical nutrition applications [14]. As demand for plant-based foods increases, its low allergenic potential further expands its use in dietary formulations for individuals with food allergies, gastrointestinal sensitivities, or specific clinical needs.

In particular, low-glutelin rice varieties have attracted attention as food-based strategies for protein-controlled diets in patients with chronic kidney disease (CKD) [19]. These low-protein rice varieties may reduce nitrogen burden while maintaining dietary familiarity in rice-dependent populations, particularly in low- and middle-income countries. Their use may also improve adherence to nutritional interventions by providing culturally accepted staple foods with modified protein content. These findings further support the potential of rice protein in personalized nutrition and clinical dietary management.

The absence of gluten further expands the applicability of rice protein in specialized nutrition. As the prevalence of celiac disease, non-celiac gluten sensitivity, and gluten-free diets increases, the demand for compatible protein ingredients has also grown. Rice protein has therefore been increasingly incorporated into gluten-free foods, nutritional supplements, and medical nutrition products, improving protein content and nutritional quality in formulations often deficient in protein and essential amino acids. Nevertheless, further studies are needed to evaluate its long-term nutritional effects, protein utilization, and clinical outcomes in sensitive populations, supporting evidence-based recommendations for personalized nutrition strategies.

### Rice Protein and Preventive Nutrition

In addition to its nutritional value, rice protein may contribute to preventive nutrition strategies aimed at reducing the risk of chronic non-communicable diseases. Plant-based dietary patterns rich in high-quality proteins have been associated with improved metabolic health, glycemic regulation, reduced cardiovascular risk, and enhanced satiety [13]. However, these benefits have largely been demonstrated for plant-based dietary patterns rather than for rice protein alone. Therefore, its contribution to preventive nutrition should be interpreted within the context of balanced diets and healthy lifestyles.

Due to its favorable digestibility and low allergenicity, rice protein may support dietary interventions for obesity, diabetes, and healthy aging, particularly in populations requiring well-tolerated protein sources. Rice-derived bioactive peptides have demonstrated antioxidant and antihypertensive activities that may reduce oxidative stress and support cardiovascular health, reinforcing the potential of rice protein as a functional ingredient for preventive nutrition strategies [17]. However, most evidence is still derived from* in vitro* studies, animal models, and mechanistic investigations, emphasizing the need for well-designed human clinical studies to confirm these effects under real dietary conditions.

Despite these advantages, important limitations remain. The relatively low lysine content of rice protein may restrict its use as a sole protein source, particularly in populations with higher protein requirements [20]. Combining rice protein with legume proteins can improve amino acid balance and overall nutritional quality. In addition, variations among rice cultivars, processing conditions, and protein fractions affect digestibility, functionality, and nutritional performance, limiting comparisons among studies and highlighting the need for standardized evaluation methods [19]. Future research should optimize protein quality through breeding, protein blending, and advanced processing technologies while expanding clinical evidence on long-term health effects and nutritional outcomes.

From a preventive nutrition perspective, the greatest potential of rice protein lies in the combination of high digestibility, low allergenicity, sensory acceptability, and compatibility with specialized dietary formulations. These characteristics make it a promising multifunctional ingredient for populations with specific dietary needs while supporting sustainable food production and health-oriented food design. However, its broader application will depend on continued advances in protein quality optimization, clinical validation, and the development of food products that combine nutritional efficacy with consumer acceptance. As interest in personalized nutrition, healthy aging, and sustainable dietary patterns continues to grow, rice protein is expected to play an increasingly important role in next-generation food systems that simultaneously address nutritional, health, and sustainability goals.

### Health-promoting Potential of Rice Protein and Derived Peptides

**Fig. 3:**
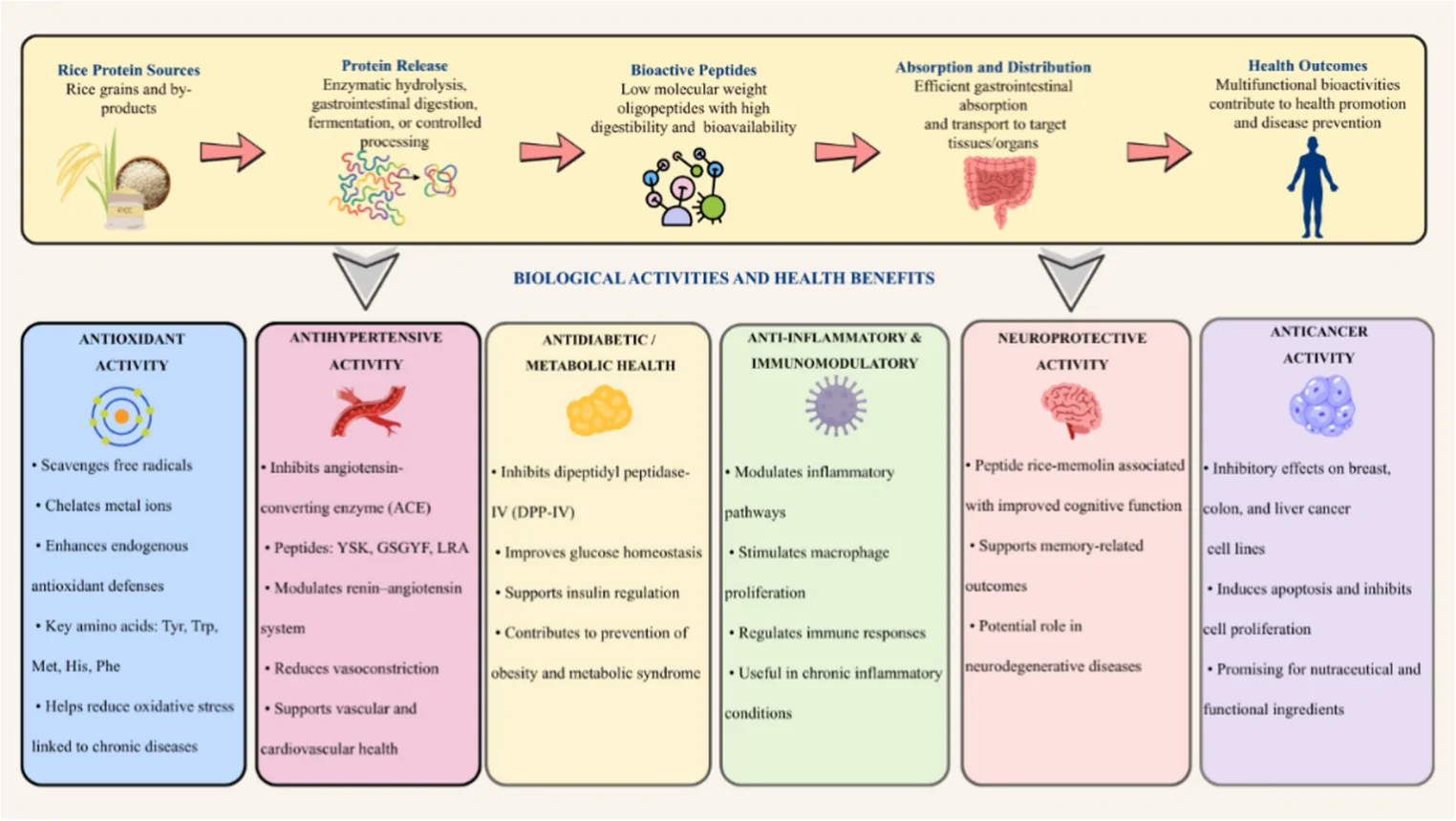
Production of rice-derived bioactive peptides and their multifunctional biological activities

Rice protein and its bioactive peptides have attracted increasing attention for their potential applications in preventive nutrition, functional foods, and health-oriented dietary strategies. Rice-derived peptides exhibit diverse biological activities, including antioxidant, antihypertensive, antidiabetic, anti-inflammatory, immunomodulatory, and anticancer effects [21, 22]. An overview of their production pathways and biological activities is presented in Fig. 3, while representative studies describing their mechanisms of action and potential health applications are summarized in Supplementary Material 2 (Online Resource 1). These properties highlight the potential of rice protein hydrolysates and oligopeptides as ingredients for nutraceuticals and functional foods targeting chronic disease prevention and health promotion.

### Bioactive Peptides, Antioxidant Potential and Anti-inflammatory Effects

Bioactive peptides are inactive within the native protein structure and are released during gastrointestinal digestion, enzymatic hydrolysis, fermentation, or controlled food processing [21]. Rice proteins are suitable precursors for bioactive peptide production because of their favorable amino acid composition, high digestibility, and abundance of sulfur-containing amino acids. Rice-derived oligopeptides also exhibit low molecular weight and favorable gastrointestinal absorption, supporting their bioavailability and biological activity. These characteristics make rice protein a promising source of functional ingredients for foods and nutraceuticals. However, the relationships among peptide structure, gastrointestinal stability, bioavailability, and physiological efficacy remain incompletely understood, highlighting the need for further research [23].

Antioxidant activity is one of the most extensively investigated biological properties of rice-derived peptides. As oxidative stress is closely associated with chronic non-communicable diseases, including cardiovascular disorders, diabetes, neurodegenerative diseases, and cancer [21], rice-derived peptides have demonstrated free radical scavenging, metal-chelating, and antioxidant defense-modulating activities. Their antioxidant capacity has been associated with hydrophobic and aromatic amino acid residues, including Tyr, Trp, Met, His, and Phe, supporting the identification of peptide sequences with enhanced bioactivity. Rice bran-derived peptide hydrolysates have also shown cytoprotective effects against oxidative stress in Caco-2 and HepG2 cells while maintaining antioxidant activity after simulated gastrointestinal digestion, suggesting potential biological activity under physiologically relevant conditions [22]. However, most evidence is based on in vitro assays, and antioxidant activity does not necessarily translate into clinical benefits, highlighting the need for further validation in human studies [23].

Low molecular weight peptides exhibit enhanced antioxidant activity and bioavailability, supporting their application in functional foods and nutraceuticals. In addition to their antioxidant properties, rice-derived peptides have demonstrated anti-inflammatory activity by modulating inflammatory pathways and oxidative stress-related responses [21]. Recent peptidomic studies identified novel peptides capable of suppressing pro-inflammatory cytokine production and regulating MAPK signaling, reducing inflammatory responses in dendritic cell models and providing new insights into their mechanisms of action [24]. The coexistence of antioxidant and anti-inflammatory activities highlights the potential of rice-derived peptides for health-oriented food formulations. However, challenges related to peptide stability during processing and storage, interactions with food matrices, and the maintenance of biological activity after gastrointestinal digestion must be addressed to enable their successful application in functional foods and nutraceuticals [23].

### Cardiometabolic, Immunomodulatory, Neuroprotective, and Osteoprotective Effects

Rice-derived peptides have demonstrated antihypertensive potential, primarily through angiotensin-converting enzyme (ACE) inhibitory activity. As hypertension is a major risk factor for cardiovascular disease, food-derived bioactive compounds have attracted increasing interest for blood pressure regulation. Several rice-derived oligopeptides, including YSK, GSGYF, and LRA, exhibit ACE inhibitory activity and antihypertensive effects in experimental models and clinical studies [23]. These peptides may promote vascular health by modulating the renin–angiotensin system and reducing vasoconstriction, supporting their use in functional foods and nutraceuticals for cardiovascular health.

Rice-derived peptides have also attracted attention for their antidiabetic potential. Several oligopeptides from rice protein hydrolysates exhibit dipeptidyl peptidase-IV inhibitory activity, contributing to glycemic control and type 2 diabetes management [23]. In addition, peptides from rice wine lees proteins have shown α-glucosidase inhibitory activity, antioxidant properties, and improved insulin resistance by modulating insulin signaling pathways, highlighting their potential for glycemic regulation [25]. Similarly, rice protein hydrolysates incorporated into bakery products reduced the predicted glycemic index and released peptides with α-amylase- and dipeptidyl peptidase-IV-inhibitory activities during digestion, suggesting complementary mechanisms for postprandial glucose control [26]. However, most evidence is derived from in vitro and animal studies, emphasizing the need for clinical trials to confirm their efficacy in diabetes prevention and management.

Beyond glycemic control, rice-derived peptides may also provide broader metabolic benefits for obesity management. In a high-fat diet-induced obesity model, the peptides IQP, VEP, and IPQ reduced adipose tissue accumulation, improved insulin sensitivity, attenuated hepatic steatosis, and decreased chronic inflammation. These effects were associated with AMP-activated protein kinase (AMPK) activation and gut microbiota modulation, suggesting that rice-derived peptides influence metabolism through complementary cellular and microbiome-mediated mechanisms [27]. These findings indicate that their physiological effects extend beyond single molecular targets and may contribute to systemic metabolic regulation.

In addition to cardiometabolic effects, rice-derived peptides have demonstrated immunomodulatory and anti-inflammatory activities, including stimulation of macrophage proliferation, modulation of immune responses, and attenuation of inflammatory pathways associated with chronic diseases [23]. Emerging evidence also indicates neuroprotective and anticancer potential. Rice-memolin, a rice bran-derived peptide, improved cognitive function and memory in experimental studies [21], while a novel rice endosperm-derived peptide exhibited antidepressant-like activity through modulation of dopaminergic signaling pathways, suggesting interactions with the gut–brain axis and central nervous system [28]. In addition, peptides from rice bran hydrolysates inhibited breast, colon, and liver cancer cell lines, highlighting their potential as functional ingredients and nutraceuticals.

The biological relevance of rice-derived peptides may also extend to musculoskeletal health. A rice bran-derived peptide promoted osteogenic and suppressed adipogenic differentiation of bone marrow mesenchymal stem cells, improving bone mass, reducing marrow adiposity, and enhancing fracture healing in osteoporosis models. These effects were associated with eIF3L-mediated signaling, revealing a previously unexplored role of rice-derived peptides in bone metabolism and healthy aging [29].

These findings indicate that rice-derived peptides exert multifunctional biological effects beyond single-target activities, including cardiometabolic regulation, immune modulation, neuroprotection, bone health, and gut microbiota interactions. However, most proposed mechanisms are supported by preclinical evidence, and challenges related to bioavailability, optimal dosage, long-term safety, and clinical efficacy remain. Addressing these limitations will be essential to support the development of scientifically validated functional foods, nutraceuticals, and preventive nutrition strategies.

### Bioavailability

The health-promoting effects of rice-derived peptides are influenced by molecular weight, amino acid composition, peptide sequence, and gastrointestinal stability. Oligopeptides containing 2–10 amino acid residues generally exhibit higher intestinal absorption and bioavailability than larger peptides and free amino acids [23]. Rice oligopeptides, in particular, have demonstrated substantially greater intestinal absorption than free amino acids, highlighting peptide size as an important determinant of biological accessibility and physiological efficacy.

The biological relevance of rice-derived peptides depends not only on their intrinsic bioactivity but also on their stability during gastrointestinal digestion, absorption, and persistence after uptake. Gastrointestinal degradation and limited absorption may reduce in vivo efficacy despite promising in vitro results, making bioavailability a key determinant of physiological benefits [30]. Consequently, understanding the relationship between peptide structure and bioavailability is essential for developing peptide-based nutritional interventions.

Several factors influence the bioavailability of rice-derived peptides, including molecular size, amino acid sequence, resistance to proteolytic degradation, hydrophobicity, molecular conformation, and intestinal transport. Although low-molecular-weight peptides generally exhibit higher absorption, physiological performance cannot be predicted solely by molecular weight because of variability among peptide fractions. Therefore, understanding structure–bioavailability relationships is essential for identifying peptide sequences with enhanced absorption and biological effectiveness [23].

Recent evidence indicates that some rice-derived peptides retain biological activity after simulated gastrointestinal digestion, suggesting favorable stability and potential physiological effects after absorption. However, bioavailability remains a major limitation, as digestion, intestinal transport, metabolism, and systemic distribution determine peptide delivery to target tissues and biological efficacy [30]. Despite increasing evidence of their biological activities, important knowledge gaps remain regarding absorption kinetics, tissue distribution, metabolism, and long-term effects in humans. Most available data are derived from *in vitro* digestion models, cell culture studies, or animal experiments, requiring caution when extrapolating findings to human health outcomes.

Future research should clarify the mechanisms governing peptide absorption, transport, metabolism, and tissue distribution, while identifying structural features that enhance bioavailability. Integrating *in vitro*, *in vivo*, and clinical studies will be essential to establish stronger links between bioavailability and health-promoting effects. These advances will improve the understanding of rice-derived peptides and support their application in evidence-based preventive nutrition and health promotion strategies [21, 22].

### Technological Functionality of Rice Protein in Modern Food Systems

Beyond its nutritional value, the successful incorporation of rice protein into food formulations largely depends on its techno-functional properties, including solubility, emulsification, foaming, gelation, and interfacial behavior [31, 32]. These properties are critical for determining texture, stability, sensory quality, and overall consumer acceptance of plant-based foods, particularly in products such as dairy alternatives, meat analogues, functional beverages, bakery systems, and nutraceutical formulations.

### Solubility and Interfacial Properties

Protein solubility strongly influences other techno-functional properties, including emulsification, foaming, gelation, digestibility, and dispersion stability [31]. However, rice protein exhibits low water solubility under neutral conditions because of its high glutelin content, hydrophobic amino acid residues, hydrogen bonding, and disulfide cross-linking [31, 32]. These structural characteristics limit protein–water interactions and reduce the functionality of native rice protein in aqueous systems, making low solubility a major constraint for applications in beverages, emulsions, and dairy alternatives.

Interfacial behavior is a key determinant of rice protein functionality in food systems. As amphiphilic molecules, proteins adsorb at oil–water and air–water interfaces, stabilizing emulsions and foams through viscoelastic interfacial films [33]. However, the compact structure and limited conformational flexibility of native rice proteins impair interfacial adsorption and emulsifying performance by restricting the exposure of hydrophobic and hydrophilic domains required for stable film formation.

### Emulsifying and Foaming Properties

Rice protein has attracted attention as a plant-based emulsifier because of its hypoallergenic properties, balanced amino acid profile, and sustainability advantages [33]. It contributes to oil droplet stabilization, emulsion viscosity, and physical stability, although its native emulsifying capacity is limited by low solubility and restricted molecular mobility. Modification strategies, including enzymatic hydrolysis, pH-shifting, ultrasound, glycosylation, phosphorylation, and interactions with polysaccharides or polyphenols, have improved interfacial adsorption and emulsion stability [31, 33].

Combined physical treatments, including pH-shifting, ultrasound, and heat processing, significantly improve the emulsifying stability and interfacial functionality of rice proteins. Under alkaline conditions, protein unfolding and aggregate dissociation increase molecular flexibility and the exposure of hydrophilic and hydrophobic groups, enhancing adsorption at oil–water interfaces [34]. These changes promote more stable oil-in-water emulsions with smaller droplet size and improved storage stability. However, maintaining colloidal stability and desirable sensory properties remains challenging in complex food matrices, particularly dairy alternatives, meat analogues, and high-moisture products, highlighting the need for further optimization of protein structuring strategies [35, 36].

Foaming properties are important for whipped products, bakery goods, aerated foods, and dairy alternatives. Ultrasonication has emerged as a sustainable strategy to improve foaming performance by inducing cavitation, particle size reduction, and protein conformational changes [37]. The increased exposure of hydrophobic groups and changes in secondary structure, including higher β-sheet and random coil content, enhance foaming capacity, foam stability, and oil-binding properties, expanding the application of rice proteins in aerated and emulsified food systems.

### Structuring Capacity and Gelation

Gelation and structuring properties are essential for incorporating rice protein into plant-based foods, particularly meat analogues and semi-solid products. Gel formation contributes to texture, water retention, viscoelasticity, and structural stability, directly influencing mouthfeel and sensory acceptance [32]. The gelation capacity of rice proteins depends on protein concentration, pH, ionic strength, thermal treatment, and intermolecular interactions, reflecting the combined effects of protein structure and processing conditions.

These structuring properties are particularly important in plant-based meat analogues, where protein network formation and water-holding capacity determine texture, juiciness, and product quality. Rice protein can contribute to hybrid protein matrices and high-moisture extrusion systems, although its native gelation capacity is generally lower than that of animal proteins. Modification strategies, particularly enzymatic hydrolysis, have improved solubility, gelation, water-holding capacity, and protein network formation while generating bioactive peptides with potential health benefits [38]. However, enhanced gelation does not always translate into optimal performance in complex food matrices, highlighting the need to balance structural modification with nutritional quality and sensory attributes.

Rheological behavior and viscoelasticity are influenced by processing-induced structural modifications. Changes in aggregation, molecular flexibility, and intermolecular interactions affect gel strength, storage modulus, and protein network organization [37]. These relationships highlight the importance of controlling protein architecture during processing to tailor texture for specific food applications. However, further studies are needed to establish structure–function relationships that accurately predict rheological performance under industrial conditions.

### Functional Modification Strategies

Among the different approaches available to tailor rice protein structure and functionality, physical and combined modification technologies have attracted considerable attention because of their ability to induce controlled structural rearrangements without extensive chemical modification. These approaches highlight that improving rice protein functionality depends primarily on tailoring protein structure rather than applying a single modification technology.

These treatments promote protein unfolding, aggregate dissociation, exposure of hydrophilic and hydrophobic groups, and changes in intermolecular interactions, improving solubility, emulsification, foaming, and rheological properties. Under alkaline conditions, increased molecular flexibility enhances interfacial adsorption, while combined treatments produce less compact aggregates with greater surface hydrophobicity and improved solubility [34]. However, their effectiveness depends on achieving these structural changes without compromising protein stability or other functional properties.

Ultrasound-assisted treatments effectively modify protein conformation by reducing particle size, disrupting aggregates, and altering secondary structure, thereby improving hydration, foaming, and emulsifying properties [37]. In addition, interactions with polysaccharides, polyphenols, and other biopolymers through electrostatic interactions or Maillard conjugation enhance water solubility, thermal stability, antioxidant activity, and interfacial functionality [31, 39]. More recently, these strategies have been extended to self-assembled protein systems. By controlling pH, temperature, ionic strength, and solvent polarity, rice proteins can form nanoparticles, nanofibrils, conjugates, delivery systems, and high-internal-phase emulsions (HIPEs), expanding applications in bioactive encapsulation, fat replacement, texture engineering, and 3D-printed foods. These advances highlight the growing importance of engineering multifunctional colloidal structures tailored to specific food applications [39, 40].

### Emerging Applications of Rice Protein and Rice-derived Peptides in Functional Food Formulations

The growing demand for sustainable, nutritious, and functional foods has accelerated the development of formulations based on alternative proteins. Advances in processing technologies and protein engineering have expanded the role of rice protein beyond nutrition, enabling its use in foods with specific nutritional, technological, and sensory properties. Owing to its hypoallergenic nature, high digestibility, techno-functional versatility, and compatibility with modern processing technologies, rice protein has emerged as a promising ingredient for plant-based foods, functional products, and sustainable food systems [14, 35].

As summarized in Supplementary Material 2 (Online Resource 2), recent studies have considerably expanded the application of rice protein and rice-derived peptides beyond traditional protein fortification, demonstrating their versatility across diverse food categories, including gluten-free bakery products, protein bars, hybrid dairy products, confectionery, beverages, and low-glycemic foods [41–45]. Furthermore, rice protein exhibits excellent compatibility with other plant proteins, enabling the development of hybrid protein systems with improved technological and nutritional performance [17, 18].

Plant-based food alternatives represent one of the fastest-growing applications of rice protein. Increasing demand for animal product alternatives has driven research to improve texture, juiciness, flavor, nutritional value, and structural organization while reproducing the fibrous structure and sensory characteristics of animal-derived foods [35]. Rice protein has attracted attention because of its mild flavor, hypoallergenic properties, high digestibility, and nutritional complementarity with legume proteins such as soy and pea [14, 35].

Recent studies have demonstrated the potential of rice protein in textured vegetable proteins and hamburger analogues produced by thermoplastic extrusion. Blends of rice and soy proteins improve techno-functional properties, texturization, and amino acid balance through the complementary nutritional characteristics of cereal and legume proteins. Rice protein may also reduce the beany flavor of soy-based formulations while improving structural and hydration properties in plant-based products [36].

Rice protein has also shown potential for seafood alternatives. Brown rice protein-based fish cake analogues exhibited a substantially lower environmental impact than conventional surimi products while maintaining desirable gelation, emulsifying properties, and compatibility with hydrocolloids and plant oils. These findings support the use of rice protein in sustainable food systems that address nutritional, sensory, and environmental demands [33].

Beyond meat and seafood analogues, rice protein has shown versatility in other food applications. In hybrid yogurts, it has been used as a partial dairy protein substitute, although further optimization is needed to improve gel firmness and reduce syneresis [46]. Cold plasma-modified rice bran protein also improved whipping, foam stability, rheological properties, and storage stability in vegetable oil-based whipped cream [42]. In addition, fermented rice protein concentrates enhanced solubility, emulsifying capacity, and sensory acceptance in vegan protein-enriched smoothies, supporting their use in beverage formulations [47].

Rice protein has also shown value in gluten-free and starch-based products. In gluten-free bread, interactions with soybean 7 S globulin promoted gluten-like network formation, improving loaf volume, crumb structure, and sensory acceptance [44]. Rice protein enrichment also modified starch–protein interactions in rice cakes, increasing slowly digestible starch while preserving desirable technological properties [48]. In addition, rice oligopeptides incorporated into low-glycemic crispy noodles reduced starch digestibility and the predicted glycemic index through peptide–starch interactions and digestive enzyme inhibition [43].

Novel structured food systems have expanded the applications of rice proteins. Rice protein–konjac glucomannan composite hydrogels improved gel strength and viscoelasticity in gel candies [49], while bicontinuous gels from rice and pea proteins generated stable structures for dairy and meat analogues [50]. Brown rice protein has also been incorporated into antioxidant- and microalgae-enriched protein bars, improving texture stability, antioxidant capacity, microbial stability, and sensory acceptance during storage [51–53]. These studies highlight the versatility of rice protein as a platform for developing sustainable, health-oriented, and technologically advanced food products.

The rapid diversification of rice protein applications has been driven by advances in food structuring technologies. Beyond conventional extrusion, shear-cell processing and three-dimensional (3D) food printing enable precise control of protein organization, allowing the development of foods with tailored structure, texture, and nutritional composition. These technologies position rice protein as a structural biomaterial for personalized and next-generation food products, supporting the transition from conventional protein ingredients to rational food design [36, 54].

### Current Limitations and Future Research Directions

Despite growing interest in rice protein as a sustainable ingredient for functional foods, several limitations still restrict its broader application. Although advances have been made in techno-functional optimization, bioactive peptide production, and innovative food applications, important challenges remain regarding nutritional standardization, clinical validation, industrial implementation, regulatory frameworks, and long-term sustainability assessment [13, 14]. Addressing these challenges will be essential to translate laboratory advances into commercially viable ingredients and scientifically validated health-promoting products.

### Nutritional and Clinical Limitations

Although nutritional strategies such as protein blending and ingredient optimization can partially overcome compositional limitations, considerable variability in amino acid composition, digestibility, protein fractions, rice cultivars, extraction methods, and processing conditions continue to affect protein quality and functional performance while complicating the establishment of standardized quality criteria and direct comparisons among studies [19].

Protein complementation by blending rice protein with lysine-rich plant proteins, particularly pea or soy, is an effective strategy to overcome the low lysine content of rice protein. Because rice protein is rich in sulfur-containing amino acids, these blends improve the essential amino acid profile and nutritional quality while preserving its hypoallergenic and highly digestible characteristics [37]. Pea–rice protein blends provide a more balanced amino acid profile than rice protein alone, although postprandial amino acid availability depends on formulation and the relative proportions of each protein source [55]. Future research should standardize protein characterization and nutritional evaluation using internationally recognized methods, including the Digestible Indispensable Amino Acid Score (DIAAS) and the Protein Digestibility-Corrected Amino Acid Score (PDCAAS).

Although rice-derived peptides and hydrolysates have demonstrated multiple biological activities, most evidence is still derived from in vitro and animal studies [21, 22]. Human clinical data on long-term effects, dose–response relationships, bioavailability, metabolic responses, and safety remain limited. In addition, interactions among rice-derived peptides, the gut microbiota, immune modulation, and chronic disease prevention are not yet fully understood. Integrating clinical nutrition with metabolomics, peptidomics, nutrigenomics, and microbiome research will be essential to clarify the mechanisms underlying the health-promoting effects of rice proteins and their derived peptides.

### Industrial and Commercial Barriers

Several industrial and commercial barriers still limit the large-scale adoption of rice protein ingredients. Variability in raw material quality, the absence of standardized extraction and modification protocols, and the relatively high costs associated with protein recovery and processing continue to restrict industrial competitiveness compared with more established plant protein ingredients [14, 18]. Moreover, comprehensive techno-economic analyses evaluating production efficiency, process costs, and industrial competitiveness remain scarce, limiting the assessment of commercial feasibility under different manufacturing scenarios [35].

Beyond technological performance, successful commercialization will depend on consumer acceptance and regulatory harmonization. Clean-label products must balance desirable texture, flavor, appearance, and nutritional quality with manufacturing efficiency, product consistency, and economic viability [36]. In addition, differences in international regulations for novel protein ingredients, functional foods, and health claims may delay market adoption, highlighting the need for closer integration among scientific research, industry, and regulatory agencies.

### Emerging Research Opportunities

Recent advances in food biotechnology, omics sciences, and protein engineering have expanded the potential applications of rice proteins and rice-derived peptides in sustainable food systems. Future research should focus on developing integrated ingredient systems that combine nutritional quality, technological functionality, health benefits, and sustainability. Promising directions include precision food design, peptide engineering, targeted delivery systems, personalized nutrition, and multifunctional protein ingredients [22, 39].

Artificial intelligence, machine learning, and predictive modeling represent emerging tools for rice protein research. These approaches can accelerate ingredient development by optimizing protein extraction, modification, processing, and formulation while reducing experimental time and resource use. They may also predict structure–function relationships and support the design of rice protein ingredients tailored to specific food applications and consumer needs.

Proteomics, peptidomics, metabolomics, and gut microbiome research may identify novel bioactive compounds and clarify the interactions between rice proteins and human health. Integrating these approaches with clinical studies, life cycle assessment, and techno-economic analyses will improve understanding of the relationships among protein structure, biological activity, industrial feasibility, and environmental sustainability. This multidisciplinary approach will support the development of scientifically validated, economically competitive, and personalized functional foods with enhanced bioavailability and targeted health benefits.

## Conclusion

The global transition toward sustainable, health-oriented, and resilient food systems has reinforced the strategic importance of rice protein beyond its role as an alternative plant protein source. Unlike previous reviews that addressed isolated aspects of rice protein, this review provides an integrated perspective linking nutritional quality, technological functionality, health-promoting potential, sustainability, and emerging food applications. The evidence demonstrates that rice protein is a multifunctional ingredient capable of supporting nutrition, food functionality, and sustainable production. Recent advances in protein modification, bioactive peptide production, and food processing have further expanded its applications in functional foods, plant-based products, and preventive nutrition. However, broader commercialization requires overcoming challenges related to protein quality standardization, clinical validation, industrial scalability, and regulatory and techno-economic frameworks.f Addressing these limitations will be essential to translate experimental advances into practical food applications. Future progress will depend on multidisciplinary research integrating food chemistry, process engineering, nutrition, biotechnology, omics sciences, computational modeling, and sustainability assessment to better understand structure–function–health relationships and accelerate the development of scientifically validated and economically competitive rice protein ingredients. These advances position rice protein as a strategic component of next-generation food systems, with the potential to contribute simultaneously to food security, preventive nutrition, and the transition toward more sustainable and resilient global diets.

## Supplementary Information

Below is the link to the electronic supplementary material.

Supplementary Material 1 (DOCX 13.7 KB)

Supplementary Material 2 (DOCX 27.3 KB)

## Data Availability

No datasets were generated or analysed during the current study.
