# Supplementary material for: Rice Protein as an Alternative Ingredient for Sustainable Food Formulations: Nutritional Quality, Functional Properties, Health-promoting Potential, and Emerging Applications
Source: Plant Foods Hum Nutr. 2026 Aug 24;81(3):113. doi: 10.1007/s11130-026-01561-6 (PMC13503404; doi:10.1007/s11130-026-01561-6)
Supplement: Supplementary file 1 [file 11130_2026_1561_MOESM1_ESM.docx]

**Rice Protein as an Alternative Ingredient for Sustainable Food Formulations: Nutritional Quality, Functional Properties, Health-Promoting Potential, and Emerging Applications**

Silvia Leticia Rivero Meza^a*^, Larissa Alves Rodrigues^a^, Sonia Medina^b^, Isabel Egea^c^, Patricia Sinnecker^d^, Mauricio de Oliveira^a*^

^a^ Department of Agroindustry Science and Technology, Federal University of Pelotas, 96010-900, Pelotas, Rio Grande do Sul, Brazil.

^b^ Laboratorio de Fitoquímica y Alimentos Saludables (LabFAS), CSIC, CEBAS, Campus Universitario de Espinardo, Edificio 25, 30100 Murcia, Spain.

^c^ Department of Stress Biology and Plant Pathology, CEBAS-CSIC, 30100 Espinardo-Murcia, Spain.

^d^ Department of Pharmaceutical Sciences, Pharmaceutical Inputs and Food Sector, Federal University of São Paulo, 09913-030, Diadema, São Paulo, Brazil.

*Corresponding authors at: mauricio@labgraos.com.br (M. de Oliveira) and silvialrmezaufpel@gmail.com (S. L. R. Meza)

**Supplementary material 1**

**Procedure and Data Analysis**

This review discusses the current scientific advances regarding rice protein as a sustainable alternative ingredient for next-generation food systems, emphasizing its nutritional relevance, techno-functional properties, health-promoting potential, and emerging applications in functional food formulations. The article integrates information related to protein quality and digestibility, hypoallergenic and gluten-free characteristics, bioactive peptides and preventive nutrition, techno-functional behavior, protein modification strategies, self-assembly systems, and applications in plant-based foods, nutraceuticals, and sustainable dietary systems. In addition, sustainability aspects associated with rice by-product valorization, circular bioeconomy approaches, advanced food structuring technologies, and future perspectives for rice protein incorporation into health-oriented food systems are critically discussed.

The studies included in this review were obtained through searches conducted in scientific databases including Web of Science, covering publications from 2022 to 2026. The keywords and search terms used included: “rice protein”, “rice bran protein”, “rice-derived peptides”, “bioactive peptides”, “plant-based proteins”, “alternative proteins”, “hypoallergenic proteins”, “gluten-free proteins”, “protein digestibility”, “techno-functional properties”, “protein modification”, “emulsification”, “gelation”, “food structuring”, “plant-based meat analogues”, “functional foods”, “nutraceuticals”, “sustainable food systems”, “circular bioeconomy”, “rice by-products”, “protein hydrolysates”, “preventive nutrition”, “self-assembly”, and “high-internal-phase emulsions”. Additional combinations involving terms such as “antioxidant activity”, “antihypertensive peptides”, “metabolic health”, “protein functionality”, “sustainability”, and “food applications” were also applied. After a comprehensive and up-to-date literature screening, 55 studies directly related to the nutritional, technological, functional, sustainability-related, and health-promoting applications of rice proteins and rice-derived peptides were selected and critically analyzed to support the development of this review.
