# Supplementary material for: Rice Protein as an Alternative Ingredient for Sustainable Food Formulations: Nutritional Quality, Functional Properties, Health-promoting Potential, and Emerging Applications
Source: Plant Foods Hum Nutr. 2026 Aug 24;81(3):113. doi: 10.1007/s11130-026-01561-6 (PMC13503404; doi:10.1007/s11130-026-01561-6)
Supplement: Supplementary file 2 [file 11130_2026_1561_MOESM2_ESM.docx]

**Rice Protein as an Alternative Ingredient for Sustainable Food Formulations: Nutritional Quality, Functional Properties, Health-Promoting Potential, and Emerging Applications**

Silvia Leticia Rivero Meza^a*^, Larissa Alves Rodrigues^a^, Sonia Medina^b^, Isabel Egea^c^, Patricia Sinnecker^d^, Mauricio de Oliveira^a*^

^a^ Department of Agroindustry Science and Technology, Federal University of Pelotas, 96010-900, Pelotas, Rio Grande do Sul, Brazil.

^b^ Laboratorio de Fitoquímica y Alimentos Saludables (LabFAS), CSIC, CEBAS, Campus Universitario de Espinardo, Edificio 25, 30100 Murcia, Spain.

^c^ Department of Stress Biology and Plant Pathology, CEBAS-CSIC, 30100 Espinardo-Murcia, Spain.

^d^ Department of Pharmaceutical Sciences, Pharmaceutical Inputs and Food Sector, Federal University of São Paulo, 09913-030, Diadema, São Paulo, Brazil.

*Corresponding authors at: mauricio@labgraos.com.br (M. de Oliveira) and silvialrmezaufpel@gmail.com (S. L. R. Meza)

**Supplementary material 2**

**Online Resource 1.** Representative studies on rice-derived bioactive peptides, their biological activities, mechanisms of action, and potential health applications.

| **Rice protein/peptide source** | **Identified bioactive peptide(s)** | **Main biological activity(ies)** | **Key mechanism(s)** | **Potential health application(s)** | **References** |
| --- | --- | --- | --- | --- | --- |
| Rice bran | RBAP (KHNRGDEF) | Osteogenic, anti-osteoporotic | Promoted osteogenic differentiation and inhibited adipogenic differentiation of bone marrow mesenchymal stem cells through eIF3L signaling; improved bone mass, reduced marrow adiposity, and accelerated fracture healing. | Osteoporosis prevention and treatment; healthy aging; bone regeneration | [1] |
| Dough-stage rice bran hydrolysate | Low-molecular-weight peptide fraction (≤3 kDa) | Antioxidant, antihypertensive, antidiabetic | Simulated gastrointestinal digestion enhanced antioxidant activity together with ACE- and DPP-IV-inhibitory activities; hydrolysates also exerted cytoprotective effects in Caco-2 and HepG2 cells. | Oxidative stress management, hypertension and glycemic control | [2] |
| Rice protein | IQP, VEP, IPQ | Anti-obesity, antidiabetic, anti-inflammatory | Activated AMPK, improved insulin sensitivity, reduced hepatic steatosis and chronic inflammation, and modulated gut microbiota composition and microbial metabolites. | Obesity, metabolic syndrome and type 2 diabetes prevention | [3] |
| Rice dreg protein | PHP1 (IPLYQHIANLAGNK), GAP1, GEP1, GAP2, OP1 | Anti-inflammatory, immunomodulatory | Twelve anti-inflammatory peptides were identified; PHP1 exhibited the strongest activity by suppressing IL-17/MAPK signaling, regulating inflammatory genes, and reducing pro-inflammatory cytokines. | Chronic inflammatory diseases and immune regulation | [4] |
| Rice wine lees protein hydrolysate | LLGDV, LGDEPL | Antidiabetic, antioxidant | Inhibited α-glucosidase activity, improved insulin resistance, exhibited antioxidant activity, and interacted with proteins involved in insulin-signaling pathways according to molecular docking and dynamics analyses. | Glycemic control and diabetes management | [5] |
| Rice endosperm protein | REAP (QQFLPEGQSQSQK) | Antidepressant-like, neuroprotective | Oral administration activated dopaminergic signaling and produced antidepressant-like effects in animal models, representing the first orally active antidepressant peptide identified from rice proteins. | Mental health and gut–brain axis modulation | [6] |
| Rice protein hydrolysate | YSK, VGRNMDE, and DPP-IV inhibitory peptides | Antidiabetic, antihypertensive | VGRNMDE exhibited α-amylase inhibitory activity, whereas YSK was identified as an ACE-inhibitory peptide. Rice protein hydrolysate also released peptides with DPP-IV inhibitory activity during digestion, contributing to reduced predicted glycemic index and improved postprandial glucose regulation. | Dietary management of postprandial glycemia, hypertension, and type 2 diabetes | [7] |

**Online Resource 2.** Representative studies reporting the incorporation of rice protein and rice-derived peptides into food products and their technological and nutritional outcomes

| **Food product** | **Rice ingredient** | **Purpose of incorporation** | **Main technological and nutritional outcomes** | **References** |
| --- | --- | --- | --- | --- |
| Plant-based seafood analogue (fish cake) | Brown rice protein isolate | Develop a sustainable seafood analogue and evaluate environmental performance | Brown rice protein enabled the production of plant-based fish cakes with substantially lower carbon footprint than conventional surimi products while maintaining technological feasibility. | [8] |
| Plant-based cheese analogue | Brown rice, broken rice, and rice bran proteins | Evaluate different rice protein fractions as cheese ingredients | Different rice protein fractions produced distinct textural properties; broken rice protein improved meltability, brown rice protein increased firmness, and rice bran protein reduced oil separation, demonstrating formulation-specific functionality. | [9] |
| Orange beverage | Jasmine rice bran protein hydrolysate | Functional beverage fortification | Optimization of sucrose, salt and citric acid successfully masked bitterness and undesirable volatiles, resulting in high consumer acceptance while incorporating bioactive peptides into the beverage. | [10] |
| Protein bars | Brown rice protein | Improve nutritional quality and storage stability | Addition of elderberry extract and *Diospyros lotus* concentrate reduced hardness, increased antioxidant activity, improved microbial stability, and enhanced sensory acceptance. | [11] |
| Vegetable oil-based whipped cream | Rice bran protein | Dairy alternative formulation | Cold plasma-modified rice bran protein improved whipping performance, foam stability, rheological behavior, overrun, texture, and storage stability by enhancing protein functionality. | [12] |
| Low-glycemic crispy noodles | Rice oligopeptides | Develop low-glycemic staple foods | Rice peptides formed starch–peptide complexes that reduced starch digestibility, lowered the predicted glycemic index, and improved the nutritional functionality of crispy noodles. | [13] |
| Plant-based hamburger analogue | Rice protein concentrate | Partial replacement of soy protein | Replacement of 10–20% soy protein with rice protein concentrate under optimized extrusion conditions improved texturization and produced textured vegetable protein suitable for hamburger analogues. | [14] |
| Hybrid yogurt | Rice protein isolate | Partial replacement of dairy proteins | Rice protein was successfully incorporated into hybrid yogurts, although optimization is still required to improve gel strength, reduce syneresis, and enhance texture stability. | [15] |
| Protein bars | Brown rice flour/protein | Improve quality during storage | Arabic and Persian gums reduced hardening, improved texture, microbial stability, and sensory quality while maintaining desirable structural properties during storage. | [16] |
| Protein bars | Brown rice protein | Nutritional and antioxidant fortification | Spirulina and phycocyanin improved antioxidant capacity, reduced hardness, modified protein secondary structure, and enhanced sensory quality. | [17] |
| Rice bread (gluten-free) | Rice proteins combined with soybean 7S globulin | Improve gluten-free bread quality | Protein interactions promoted gluten-like network formation, resulting in lower hardness, greater porosity, improved springiness, and higher sensory acceptance. | [18] |
| Gel candy | Rice protein/konjac glucomannan hydrogel | Develop novel plant protein hydrogel | Composite hydrogel improved gel strength, cohesiveness, viscoelasticity, and microstructure, providing an alternative strategy for confectionery applications. | [19] |
| Plant-based gels | Rice and pea proteins | Develop structured vegan foods | Rice protein–pea protein–gellan gum systems formed stable bicontinuous gels with tunable texture, water retention, and rheological properties for vegan food applications. | [20] |
| Vegan smoothie | Fermented rice protein concentrate | Protein fortification | Solid-state fermentation enhanced protein solubility, emulsifying and foaming properties, water/oil holding capacity, and sensory acceptance, enabling successful incorporation into vegan smoothies. | [21] |
| Rice cakes | Rice protein | Nutritional enrichment | Rice protein supplementation modified starch–protein interactions, increased slowly digestible starch, enhanced thermal stability, and improved protein quality while maintaining product characteristics. | [22] |

**References**

1. Yin JJ, Zou Y, Yao K, Gao P, Zhong W, Zhang XH, Wang L (2026) Unlocking the potential of rice bran protein: modification strategies and functional enhancements. Curr Res Food Sci 12:101335. <https://doi.org/10.1016/j.crfs.2026.101335>

2. Khongla C, Musika S, Luasiri P, Katemala S, Laosam P, Thawornkuno C, Kosinan A, Suwanangul S, Sangsawad P (2026) Dough grain rice bran valorization via semi-pilot scale papain hydrolysis: β-turn enriched bioactive peptides with cellular antioxidant activity, GI-enhanced ACE/DPP-IV inhibition, and cookie application. J Agric Food Res 27:102869. <https://doi.org/10.1016/j.jafr.2026.102869>

3. Liu L, Jian J, Yu S, Li S, Wang J, Bu T, Wu J (2025) Rice protein-derived hypoglycemic peptides IQP, VEP and IPQ alleviate obesity-related metabolic disorders in high-fat diet (HFD)-induced C57BL/6J mice through activation of AMPK and modulation of gut microbiota and related metabolites. Food Biosci 72:107427. <https://doi.org/10.1016/j.fbio.2025.107427>

4. Qu T, Wu Y, Zhang Z, Chen Z, Wu H, Mu D, Xu Z, Chen ML, Cheng Y, Tan W, Xie Y, Huang Q, Hu J, Wen L (2025) From by-product to biopeptide: Insight into the anti-inflammatory mechanism of peptides from rice dreg via peptidomics, multi-in silico, and *in vitro* approaches. J Future Foods. <https://doi.org/10.1016/j.jfutfo.2025.07.008>

5. Ding L, Xiong W, Guo L, Zheng X, Cai S (2024) The *in vitro* anti-diabetic and antioxidant properties of protein hydrolysate from rice wine lees and its main novel bioactive peptides: Revealing the interaction with proteins of insulin signaling pathways in silico. Food Biosci 62:105562. <https://doi.org/10.1016/j.fbio.2024.105562>

6. Asakura S, Kaneko K, Kawano K, Shobako M, Xu C, Sato M, Kurabayashi A, Suzuki H, Ito A, Higuchi Y, Nakayama R, Takahashi H, Ohinata K (2024) Characterization of rice endosperm-derived antidepressant-like peptide (REAP): An orally active novel tridecapeptide derived from rice protein. Peptides 177:171184. <https://doi.org/10.1016/j.peptides.2024.171184>

7. Oliviero V, Tagliamonte S, Ferracane R, Vitaglione P (2023) Potential antidiabetic effect of muffins formulated with different protein hydrolysates: Role of bioactive peptides formed during digestion. J Funct Foods 109:105810. <https://doi.org/10.1016/j.jff.2023.105810>

8. Zhao L, Sharratt PN, Du J (2026) Assessing the carbon footprint of brown rice protein-based seafood analogue using a life cycle assessment approach. Future Foods 13:100959. <https://doi.org/10.1016/j.fufo.2026.100959>

9. Galib RM, Rahman MM (2026) Three shades of plant protein from a single rice cultivar: Insights into subunit profiles, molecular structures, functional and nutritional properties, and cheesemaking performance. Future Foods 13:100875. <https://doi.org/10.1016/j.fufo.2025.100875>

10. Hunsakul K, Laokuldilok T, Prinyawiwatkul W, Pakakaew P, Posri W, Utama-ang N (2026) Effects of sucrose, salt and citric acid mixture in Jasmine rice bran protein hydrolysate on volatile compounds and application in an orange beverage. J Future Foods 13:101014. <https://doi.org/10.1016/j.fufo.2026.101014>

11. Kaspour M, Pourfarzad A, Goli SAH (2026) Structural optimization of whey–brown rice protein bars with elderberry and *Diospyros lotus* extracts using FTIR and multivariate analysis. LWT 250:119438. <https://doi.org/10.1016/j.lwt.2026.119438>

12. Li L, Liu C, Wang N, Yu D, Wang L, Zhang N (2026) Mechanistic insights into cold plasma treatment of rice bran protein for enhanced vegetable oil-based whipped cream properties. Food Hydrocoll 172:112106. <https://doi.org/10.1016/j.foodhyd.2025.112106>

13. Sun A, Zheng S, Chen M, Chen X, Li Y, Liang Z, Guo X, Zhu J (2026) Fabrication of low-digestible potato starch–rice peptide complexes via heat-moisture treatment for low-glycemic-index crispy noodles. LWT 252:119587. <https://doi.org/10.1016/j.lwt.2026.119587>

14. Cruz BRG, Sebastiao VG, Teixeira RHF, Silva JF, Pollonio MAR, Sato ACK, Steel CJ (2025) Innovative hamburger analogs: Partial soy replacement with rice protein and moisture optimization in extruded textured vegetable protein ingredient. ACS Food Sci Technol 5:1258-1267. <https://doi.org/10.1021/acsfoodscitech.4c00677>

15. Méndez-Galarraga MP, Curutchet A, Budelli E, Oliveira-Rizzo C, Di Paolo A, Rodríguez Arzuaga M (2025) Microbiological and physical evaluation of nonfat set-type hybrid yogurts formulated with soy and rice proteins. Future Foods 12:100835. <https://doi.org/10.1016/j.fufo.2025.100835>

16. Movaghar S, Pourfarzad A, Nikoo AM (2025) Optimization of brown rice protein bars formulated with Arabic and Persian gums: Insights from physicochemical, FTIR, and chemometric analyses. LWT 231:118303. <https://doi.org/10.1016/j.lwt.2025.118303>

17. Pourghasemian P, Pourfarzad A, Babakhani A (2025) Optimization of a brown rice protein bar supplemented with *Spirulina* and phycocyanin: FTIR-based analysis of protein secondary structures and multivariate quality assessment. Appl Food Res 5:101259. <https://doi.org/10.1016/j.afres.2025.101259>

18. Yang Y, Wu MQ, Ma CM, Xu Y, Zhu PY, Wang B, Zhang G, Liu XF, Zhang N (2025) Soybean 7S globulin and rice proteins form network structures: A comprehensive study of rice dough and rice bread. Food Chem 493:145805. <https://doi.org/10.1016/j.foodchem.2025.145805>

19. Zhang J, Li X, Xiong W, Yao Y, Mo C, Cao W, Wang L (2025) Construction of alkali-induced rice protein/konjac glucomannan hydrogel and its application in gel candy. Food Chem 496:146780. <https://doi.org/10.1016/j.foodchem.2025.146780>

20. Ozorio L, Corradi AP, Perrechil F (2024) Pea and rice proteins with gellan gum and monovalent salts for the development of plant-based gels for food applications. Appl Food Res 4:100601. <https://doi.org/10.1016/j.afres.2024.100601>

21. Patil ND, Kumar A, Sridhar K, Chawla P, Sharma M (2024) Enhancing functional attributes of rice protein concentrate through *Aspergillus awamori* MTCC 6652 assisted solid-state fermentation: Development of value-added vegan smoothie. Food Bioprod Process 147:11–26. <https://doi.org/10.1016/j.fbp.2024.06.001>

22. **Liu M, Hu H, Ouyang K, Li G, Chen Y, Wang S, Zhang Q, An J, Yu L, Zhao Q (2025) Effects of rice, soy, and whey protein additions on quality characteristics and** in vitro **digestibility of rice cakes.** Int J Biol Macromol **320:145775.** <https://doi.org/10.1016/j.ijbiomac.2025.145775>
